# Antimony(V) and Bismuth(V) Complexes of Lapachol: Synthesis, Crystal Structure and Cytotoxic Activity

**DOI:** 10.3390/molecules161210314

**Published:** 2011-12-13

**Authors:** Ludmila G. de Oliveira, Meiriane M. Silva, Flávia C. S. de Paula, Elene C. Pereira-Maia, Cláudio L. Donnici, Carlos A. de Simone, Frédéric Frézard, Eufrânio N. da Silva Júnior, Cynthia Demicheli

**Affiliations:** 1 Departamento de Química, Instituto de Ciências Exatas, Universidade Federal de Minas Gerais (UFMG), Av. Antônio Carlos 6627, 31270-901 Belo Horizonte, MG, Brazil; 2 Laboratório de Química Sintética e Heterocíclica, Universidade Federal de Minas Gerais (UFMG), 31270-901 Belo Horizonte, MG, Brazil; 3 Departamento de Física e Informática, Instituto de Física, Universidade de São Paulo (USP), 13560-970 São Carlos, SP, Brazil; 4 Departamento de Fisiologia e Biofísica, Instituto de Ciências Biológicas, Universidade Federal de Minas Gerais (UFMG), 31270-901 Belo Horizonte, MG, Brazil

**Keywords:** antimony(V) complex, bismuth(V) complex, lapachol, cytotoxicity, cancer

## Abstract

Antimony(V) and bismuth(V) complexes of lapachol have been synthesized by the reaction of Ph_3_SbCl_2_ or Ph_3_BiCl_2_ with lapachol (Lp) and characterized by several physicochemical techniques such as IR, and NMR spectroscopy and X-ray crystallography. The compounds contain six-coordinated antimony and bismuth atoms. The antimony(V) complex is a monomeric derivative, (Lp)(Ph_3_Sb)OH, and the bismuth(V) complex is a dinuclear compound bridged by an oxygen atom, (Lp)_2_(Ph_3_Bi)_2_O. Both compounds inhibited the growth of a chronic myelogenous leukemia cell line and the complex of Bi(V) was about five times more active than free lapachol. This work provides a rare example of an organo-Bi(V) complex showing significant cytotoxic activity.

## 1. Introduction

**Figure 1:**
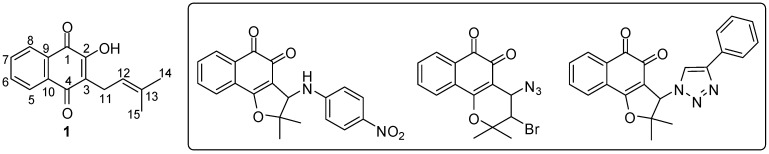
Structure of lapachol (**1**) and lapachone derivatives.

Lapachol, [2-hydroxy-3-(3′-methyl-2-butenyl)-1,4-naphthoquinone, Lp, **1**, Figure 1], is a natural product obtained from the hard core of various trees of the Bignoniaceous family. Several reports about the antitumor [1], antibiotic [2], antimalarial [3], trypanocidal [4] and leishmanicidal [5] activities of lapachol (**1**) were reported. Lapachones (Figure 1) obtained from lapachol (**1**) with potent pharmacological activities are also described [6,7], which shows the potential of compound **1** for structural modifications and synthesis of new active substances.

In general, the activity of the substances of the lapachol group is related with generation of reactive oxygen species (ROS) by redox cycling or intercalation between DNA base pairs and their electronic and redox properties are fundamental to understand their mechanism of action [8,9]. The insertion of electron-withdrawing or electron-donating groups can modulate the properties of these quinonoid compounds and represents an important strategy to obtain new active compounds [10]. Another means of modifying the activity of naphthoquinones, such as lapachol (**1**), is to form new metal complexes.

There are many reports of metal complexes with lapachol as ligand [11,12,13,14]. In a recent work, a manganese(II) lapacholate polymer was described, in which lapachol acts as a tridentate ligand, with two adjacent oxygen atoms chelating the metal and the remaining *trans*-quinonic oxygen bound to a different metal center [15].

Bismuth compounds have been used in medicine for more than two centuries for the treatment of *Helicobacter pylori* infections and other gastrointestinal disorders. Antimony compounds have been used for nearly a century in the clinical treatment of the parasitic disease, leishmaniasis. About 70 years ago, trivalent antimonial drugs were substituted by less toxic pentavalent antimonials in the treatment of this disease [16,17].

In addition, antimony and bismuth(III) organo-derivatives were evaluated for their cytotoxic activity against tumor cell lines [18]. However, organobismuth(III) compounds did not show any useful activity [18]. Silvestru *et al.* reported for the first time the antitumoral activity of organoantimony(III) derivatives [19,20,21]. The relatively high antitumoral activity of organo-antimony(V) derivatives has also been reported in literature [22,23,24]. However, to the best of our knowledge, no organobismuth(V) compound has been evaluated for antitumor activity [19,25].

The antifungal and antibacterial activities of some organobismuth(V) and organoantimony(V) compounds have also been reported [26,27]. Despite these previous studies, organoantimony and organobismuth compounds still deserve further chemical and pharmacological investigations. In the present work, we report the synthesis, characterization, and evaluation of the cytotoxic activity of two new complexes of lapachol, (Lp)(Ph_3_Sb)OH (**2**) and (Lp)_2_(Ph_3_Bi)_2_O (**3**), in an attempt to ally the activities of these metals to that of lapachol.

## 2. Results and Discussion

The reaction of Ph_3_SbCl_2_ and Ph_3_BiCl_2_ with lapachol (Lp, **1**) led to the formation of the complexes (Lp)(Ph_3_Sb)OH (**2**) and (Lp)_2_(Ph_3_Bi)_2_O (**3**) via substitution of the chlorine atoms by the oxygen atoms of the ligand lapachol (**1**).

Conductance measurements of 10^−3^ M solutions in dimethylsulfoxide of compounds **2** and **3** were 5.68 and 7.05 µS/cm, respectively, indicating the absence of ionic species in solution.

Compounds **2** and **3** suffer a displacement to lower frequency of 98 and 110 cm^−1^, respectively, when compared to the lapachol carbonyl group (1,660 cm^−1^) in the infrared spectrum. The absorption at 1,028 cm^−1^ assigned to C-O stretching vibration for compound **1** suffers a displacement to higher frequency in complexes **2** and **3** of 30 and 28 cm^−1^, respectively. In the complexes **2** and **3** the -OH stretching vibration at 3,300 cm^−1^ was not observed indicating the complexation with lapachol (**1**).

Comparisons of ^13^C-NMR data between the metal complexes **2** and **3** and substance **1** (Table 1) showed an upfield shift of 0.38 ppm for C1 and a downfield shift of −2.5 ppm for C2 in the case of compound **2**; an upfield shift of 0.38 ppm for C1 and a downfield shift of −2.5 ppm for C2 in the case of compound **3**. These shifts can be attributed to the binding of the metal to the oxygen atom linked to C1 and C2.

**Table 1:** ^1^H- and ^13^C-NMR data of lapachol (Lp, **1**) and (Lp)(Ph_3_Sb)OH (**2**) and (Lp)_2_(Ph_3_Bi)_2_O (**3**).

Compound	Lp		(2)	(3)	Lp	(2)	(3)
Solvent	CDCl_3_		CDCl_3_	CDCl_3_	CDCl_3_	CDCl_3_	CDCl_3_
Number	*δ* ^13^C		*δ* ^13^C	*δ* ^13^C	*δ* ^1^H	*δ* ^1^H	*δ* ^1^H
**1**	184.5	182.6		183.3			
**2**	152.7	158.1		156.0			
**3**	123.5	126.0		122.1			
**4**	181.7	184.9		185.5			
**5**	126.0	126.1		125.7	8.1 (*dd* 6.4 and 1.3)	8.0 (*d*, 7.6)	8.2 (*d*, 7.6)
**6**	132.8	134.3		131.5	7.7 (*td*, 6.4 and 1.3)	7.6 (*t*, 7.5)	7.7 (*t*, 7.8)
**7**	134.8	133.4		134.0	7.7 (*td*, 6.4 and 1.3)	7.5 (*t*, 7.6)	8.0 (*t*, 7.6)
**8**	126.7	126.3		125.9	8.1 (*dd*, 6.4 and 1.3)	8.1 (*d*,7.6)	8.5 (*d*, 7.6)
**9**	129.4	132.0		130.2			
**10**	132.9	133.4		133.6			
**11**	22.6	23.2		23.2	3.3 (*d*, 6.7)	3.3 (*d*, 7.2)	3.4 (*d*, 6.8)
**12**	119.6	121.3		121.9	5.2 (*m*, 6.7)	5.1 (*m*, 6.8)	5.1 (*m*, 6.8)
**13**	133.8	132.8		133.2			
**14 or 15**	25.7	25.6		25.7	1.7 *s*	1.6 *s *	1.6 *s*
**15 or 14**	17.8	17.7		17.8	1.6 s	1.5 *s*	1.7 *s*

*δ*, chemical shifts in parts per million (ppm); *J*, coupling constants in Hertz.

^1^H-NMR data also show the disappearance of the -OH signal for all compounds when compared to compound **1**, indicating the deprotonation of -OH group and its involvement in the formation of Sb-O and Bi-O bonds.

**Figure 2:**
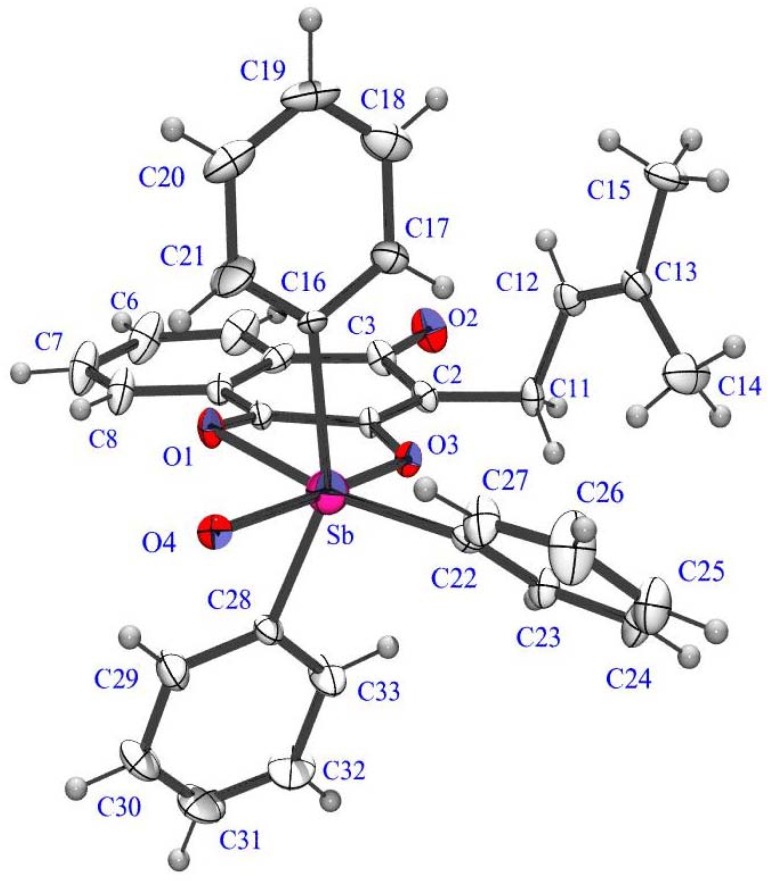
An ORTEP-3 projection of compound **2** showing the atom-numbering and displacement ellipsoids at the 30% probability level.

**Figure 3:**
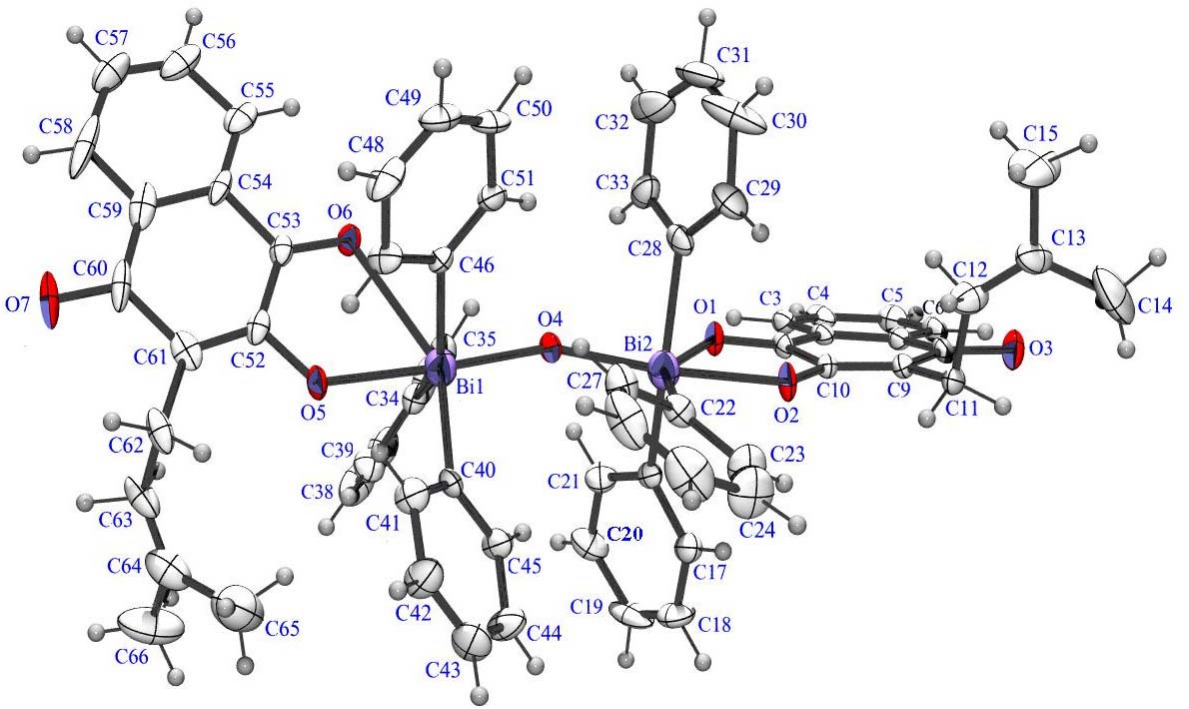
An ORTEP-3 projection of compound **3** showing the atom-numbering and displacement ellipsoids at the 30% probability level.

By X-ray analysis it is possible to observe that in the antimony(V) complex the Sb atom has a distorted-bipyramidal environment, defined by three O atoms (O1, O3, O4) and one C atom C22 at the equatorial site and two C atoms (C16, C28) at the axial site. In the bismuth(V) complex the Bi atoms are bridged by one O atom (O4) into a binuclear Bi2 subunit. The Bi atoms in both units has a distorted-bipyramidal environment, defined by three O atoms (O1, O2, O4) and C22 at the equatorial site and two C atoms (C16, C28) at the axial site for one unit and (O4, O5, O6) and C40 at the equatorial site and two C atoms (C34, C46) at the axial site for the other unit respectively. The Sb-O [2.074(4)–2.467(2) Å], Sb-C [2.118(7)–2.139(6) Å] and Bi-O [1.986(2)–2.722(7) Å], Bi-C [2.100(2)–2.126(9) Å] distances are consistent with those previously observed in the related reported complexes [28,29,30]. The ORTEP-3 diagrams of the molecules are shown in Figure 2 and Figure 3. Table 2 lists the main crystallographic parameters for both compounds.

The different complexes synthesized in this work were then compared with Lp and their respective starting materials, Ph_3_SbCl_2_ and Ph_3_BiCl_2_, for their ability to inhibit the growth of a K562 tumor cell line. The concentrations of compounds required to inhibit 50% of cell growth, IC_50_, are shown in Table 3. All lapachol complexes were able to inhibit cellular growth in a concentration dependent manner.

**Table 2:** Crystal data and structure refinement.

Empirical formula	C_66_H_58_Bi_2_O_7_		C_33_H_28_SbO_4_		
Formula weight	1381.08		610.30		
Temperature	295 (2) K		295 (2) K		
Wavelength	0.71073 Å		0.71073 Ă		
Crystal system, space group	Monoclinic, C2/c		Monoclinic, P2_1_/c		
Unit cell dimensions	a = 27.8310(5) Å		a = 14.9030(2) Å			
	b = 15.6580(3) Å	β = 100.58(1)°	b = 8.9130(3) Å	β = 99.09(1)°
	c = 27.1660(5) Å		c = 21.8200(3) Å	
Volume	11637.2(4) Å^3^		2861.9(6) Å^3^		
Z, Calculated density	4, 1.58 Mg/m^3^		4, 1.42 Mg/m^3^		
Absorption coefficient	2.406 mm^−1^		0.99 mm^−1^		
F(000)	5408		1236		
Crystal size	0.42 × 0.31 × 0.29 mm		0.32 × 0.27 × 0.22 mm		
Theta range for data collection	2.6 to 27.53°		2.6 to 27.4°		
Limiting indices	−36 ≤ h ≤ 36, −20 ≤ k ≤ 20, −35 ≤ l ≤ 35		−19 ≤ h ≤ 19, −11 ≤ k ≤ 11, −27 ≤ l ≤28		
Reflections collected/unique	68287/13271 [R_int_ = 0.052]		42238/6275 [R_int_ = 0.102]		
Completeness to theta = 27.52	98.8%		98.8%		
Refinement method	Full-matrix least-squares		Full-matrix least-squares		
Data/restraints/parameters	10165/0/676		4902/0/344		
Goodness-of-fit on F^2^	1.242		1.027		
Final R indices [I > 2σ(I) ]	R_1_ = 0.087, wR_2_ = 0.122		R_1_ = 0.080, wR_2_ = 0.098		
R índices (all data)	R_1_ = 0.232, wR_2_ = 0.289		R_1_ = 0.245, wR_2_ = 0.266		
Largest diff. peak and hole	1.401 and −2.035 e.Å^-3^		1.935 and −0.965 e.Å^−3^		

**Table 3:** Growth inhibition of K562 cells by lapachol, Ph_3_BiCl_2_, Ph_3_SbCl_2_, and their complexes.

	Lp	Ph_3_BiCl_2_	Ph_3_SbCl_2_	(Lp)_2_(Ph_3_Bi)_2_O	(Lp)(Ph_3_Sb)OH
^a^ IC_50_ (μM)	9.2 ± 0.9	30.1 ± 0.1	17.6 ± 1.6	1.8 ± 0.3	36.4 ± 1.8

^a^ IC_50_ is the concentration required to inhibit 50% of K562 cell growth (mean ± SD).

Interestingly, the triorgano Bi(V) complex exhibits an IC_50_ of 1.8 µM, whereas the starting metallic salt Ph_3_BiCl_2_ exhibits an IC_50_ of 30.0 µM. For the Sb(V) complex, the activity was not improved, presumably because of its lower stability in aqueous medium (data not shown). For the sake of comparison, the IC_50_ value determined for cisplatin under the same experimental conditions is 1.0 ± 0.4 µM. The most important result is that bismuth complex (**3**) is approximately five times more active than Lp and 17 times more than the metal starting salt, Ph_3_BiCl_2_. Thus, the coordination of triphenylbismuth by lapachol (**1**) is expected to improve its therapeutic activity and is a very attractive for pharmacological applications.

Almost all of the bismuth(V) complexes are very unstable in aqueous media. One rare example is a seven-coordinated bismuth(V) troponolate complex [31]. Its stability in aqueous media was attributed to the steric shielding of the bismuth(V) in the compound. Stable bismuth(V) compounds also require strong electronegative bonding partners. Stability studies of (Lp)_2_(Ph_3_Bi)_2_O complex exploiting its VIS absorption spectrum indicates that it does not undergo decomposition in aqueous medium (data not shown), a fact that can attributed to the oxidants and eletrophiles properties of the lapachol and the phenyl groups.

## 3. Experimental

### 3.1. General

Triphenylantimony dichloride, triphenylbismuth dichloride and lapachol (**1**) were obtained from Aldrich. Triethylamine was obtained from Sigma. All chemicals used were of reagent grade. The infrared (IR) spectra have been recorded on Perkin Elmer FTIR spectrum GX spectrometer using KBr pellets. Conductivity data were obtained with a Digimed DM31 apparatus equipped with a conductivity cell (C = 1.185 cm^−1^). Elemental analyses were carried out using a Perkin-Elmer 240 Elemental Analyzer. Atomic absorption analyses of bismuth and antimony contents were carried out on a model 8200 Hitachi atomic absorption spectrophotometer. ^1^H- and ^13^C-NMR were recorded at room temperature using a Bruker DRX400-AVANCE spectrometer, in the solvents indicated, with TMS as internal standard. Chemical shifts (*δ*) are given in ppm and coupling constants (*J*) in Hertz.

### 3.2. Cell Line, Culture and Cytotoxicity Assays

K562 is a cell line of chronic myelogenous leukemia established from pleural effusion of a 53 year-old female in terminal blast crisis, which was purchased from the Rio de Janeiro Cell Bank (number CR083 of the RJCB collection). Cells were grown in suspension in RPMI 1640 supplemented with 10% fetal calf serum in a humidified atmosphere with 5% CO_2_ at 37 °C.

In the cytotoxicity assays, 1 × 10^5^ cells/mL were incubated continuously in the absence and presence of various concentrations of tested compounds. After a 72 h culture period, cell number was determined by Coulter counter analysis. Cell viability was checked by Trypan Blue exclusion. The sensitivity to the drug was evaluated by the drug concentration needed to inhibit cell growth by 50%, the IC_50_. The mean IC_50_ ± SD was determined in three independent experiments each performed in duplicate.

### 3.3. Synthesis of the Complex (Lp)(Ph_3_Sb)OH (**2**) and (Lp)_2_(Ph_3_Bi)_2_O (**3**)

#### 3.3.1. Preparation of (Lp)(Ph3Sb)OH (**2**)

Triethylamine (70 µL) was added to a mixture of lapachol (121 mg, 0.5 mmol) and triphenylantimony(V) dichloride (212 mg, 0.5 mmol) in chloroform (20 mL). The resulting mixture was stirred for 4 h at room temperature and, after the removal of the solvent under vacuum, a solid compound was obtained. The substance was subsequently dissolved in acetone and poured into water and a precipitate was obtained. The triethylammonium hydrochloride formed was removed with water. The orange-colored compound was obtained in 75% yield and crystals suitable for X-ray analysis were prepared by vapor diffusion of petroleum ether into a dichloromethane solution of the compound. m.p. 154–156 °C. For ^1^H- and ^13^C-NMR data and the comparison between Lp (**1**) and the complex **2** see Table 1. Anal. Calc.: C, 64.82; H, 4.78; Sb, 19.90. Found: C, 65.30; H, 4.52; Sb, 20.64%. IR (KBr, cm^−1^): 1562 (ν C=O), 1641 (ν C=O), 1058 (ν C-O).

#### 3.3.2. Preparation of (Lp)_2_(Ph_3_Bi)_2_O (**3**)

Triethylamine (70 µL) was added to a mixture of lapachol (121 mg, 0.5 mmol) and triphenylbismuth(V) dichloride (192 mg, 0.5 mmol) in tetrahydrofuran (20 mL). The same procedure as described above was followed for the preparation of this complex. The substance was obtained in 79% yield, m.p. 126–129 °C. For ^1^H- and ^13^C-NMR data and a comparison between Lp (**1**) and the complex **3** see Table 1. Anal. Calc.: C, 57.40; H, 4.23; Bi, 29.98. Found: C, 57.31; H, 4.09; Bi, 29.03%. IR (KBr, cm^−1^): 1551 (ν C=O), 1624 (ν C=O), 1057 (ν C-O).

### 3.4. X-Ray Analysis

X-ray diffraction data collections for the compounds were performed on an Enraf-Nonius Kappa-CCD diffractometer (95 mm CCD camera on κ-goniostat) using graphite monochromated MoKα radiation (0.71073 Å), at room temperature. Data collection was carried out using the COLLECT software [32] up to 50° in 2θ. Final unit cells parameters were based on 36671 reflections for bismuth complex and 28720 for antimony complex, respectively. Integration and scaling of the reflections, correction for Lorentz and polarization effects were performed with the HKL DENZO-SCALEPACK system of programs [33]. The structure of compound was solved by direct methods with SHELXS-97 [34]. The models were refined by full-matrix least squares on F^2^ using SHELXL-97 [34]. The program ORTEP-3 [35] was used for graphic representation and the program WINGX [36] to prepare material for publication. All H atoms were located by geometric considerations placed (C-H = 0.93–0.96 Å) and refined as riding with U_iso_(H) = 1.5U_eq_(C-methyl) or 1.2 U_eq_ (other). Crystallographic data for compound have been deposited with the Cambridge Crystallographic Data Center as Supplementary Publication No. CCDC 834105 and CCDC 834106. Copies of the data can be obtained, free of charge, on application to CCDC, 12 Union Road, Cambridge CH21EZ, UK (Fax: +44-1223-336-033 or Email: deposit@ccdc.cam.ac.uk).

## 4. Conclusions

New triorganometal lapachol derivatives were synthesized and their crystal structure was determined. Furthermore, this work shows that coordination of triphenylbismuth by Lp (**1**) resulted in a more cytotoxic compound, when compared to either the starting metallic salt (Ph_3_BiCl_2_) or lapachol. Interestingly, this is the first example of a metal lapachol complex that displays higher cytotoxic activity than lapachol alone.
